# The Institutional Worker

**Published:** 1913-06-21

**Authors:** 


THE
### INSTITUTIONAL WORKER
**Being a Special Supplement to "The Hospital"**

### OUR BUREAU OF INFORMATION.

### Rules for Correspondents.

1. Every letter must be accompanied by the coupon to be cut from the back cover (inside page) of The Hospital, current issue, and must contain the name and address of the correspondent with pseudonym for publication if desired. Replies by post cannot be given, save under exceptional circumstances at the Editor's discretion.

2. Letters from Approved Homes in reply to special needs published in the Bureau should state terms and full particulars, and be sent prepaid under cover to the Editor of the Bureau with name written across coupon for identification.

3. Proprietors of Homes which have not yet been entered on the List of Approved Homes, but have spare accommodation likely to suit special needs, are invited to write for an application form for registration. The fee for registration, which includes two announcements of the Home in the Bureau and other privileges, is 10s.

4. All communications to be addressed to the Editor of The Hospital, 28 Southampton Street, Strand, London, W.C., and marked "Bureau of Information."

### *THE INSTITUTIONAL OFFICER.*

Contributions from officers engaged in the work of institutions for the relief of the sick and afflicted, whether voluntary or rate supported, are invited, and will be paid for in accordance with scale if made use of.

### An Opportunity for Institutional Workers.

Every enthusiastic hospital worker endeavours to keep in touch with as many institutions as possible, either by correspondence or visitation, so that he may, by a comparison of his methods with those of other workers, eliminate the weaknesses of his own institution and raise it to the highest standard of efficiency possible. Time spent in this manner is always profitable, for there is no institution, however small or obscure, that has not something to give, in the way of useful or suggestive information, to him who seeks; and it is only by this mutual exchange of ideas and experiences that the institutional officer can hope to keep abreast with the rapid developments in this sphere of work.

Unfortunately, the opportunity of the hospital officer of increasing his knowledge in this manner is often limited to an occasional visit to a neighbouring institution, or to correspondence with a dozen or so institutions when the consideration of some important matter indicates the need. Thus it is that useful knowledge upon a variety of details—the fruits of much care, thought, and ingenuity of hospital workers—is never brought to light. Yet how helpful it would be if the accumulated experiences of all the workers in this field could be disseminated for the benefit of each institution!

How Useful Knowledge may be Disseminated.

The object of "The Institutional Worker" is to centralise and distribute information of this character through its columns. Our aim can quite easily be achieved through the earnest co-operation of our readers, and a perusal of these columns should indicate the lines upon which co-operation is desired.

1. We ask our readers to communicate with us when they require our assistance, and we will spare no pains in giving them such help in connection with their work as they may require.

2. We invite every institutional worker to contribute to our columns notes upon the many technical details in connection with institutional work. We wish it to be understood that we extend this invitation to departmental workers as well as to administrative officers. The details of office management, the methods of raising funds, the management of the out-patient department, the dispensary, the kitchen and the laundry, the care of the buildings, plant and apparatus—all present their peculiar difficulties and interests, and many useful observations upon these subjects might be made.

Co-operation of Kindred Institutions.

Although in the foregoing we have more particularly referred to hospital workers, our invitation is also extended to workers in other institutions undertaking the treatment and care of the sick and needy, whether voluntary or rate-supported, for a closer co-operation of these institutions and agencies, and an exchange of experiences between their officers is much to be desired.

### *THE SICK AND DISTRESSED.*

**The Editor is prepared to make known without charge the needs of any who may be sick or in difficulties, and to guide them in making choice of Homes for treatment or convalescence. Reduced terms can often be arranged with Homes on the Approved List for those unable to pay full fees. Queries are specially invited from those who are engaged in any kind of philanthropical work. A new edition of the leaflet describing the purposes of the Bureau of Information is now ready and will be sent free of charge on application.**

### Help for Phthisical Case.

We would remind our readers of the urgent needs of this case, full particulars of which appeared in our last week's issue. Any assistance or suggestions for dealing with the case that they can give will be gratefully welcomed. Replies should be addressed to E. G., 161b.

### Permanent Home Required.

We require a permanent home for a lady suffering from a chronic illness, which does not, however, prevent her from helping herself. Her age is fifty-one. A comfortable home where she could receive such nursing attention as she requires and enjoy the society of others would meet the case. A weekly payment of 25s. can be made. Replies should be addressed to C. C., 162a.

### Home for Boys Required.

Can any of our readers suggest a suitable home or institution where two boys suffering from constant incontinence, aged nine and eleven years respectively, could be received for a small payment? As they are unable to attend the Council schools, some home instruction is essential. Replies should be addressed to H., 163a.

### Home for Epileptic Required.

A home is required, near Halifax if possible, for a young woman who is suffering from epilepsy. A payment of 10s. a week can be made towards her maintenance. If any of our readers can suggest a suitable home, or if any of our Approved Homes can accommodate this case, they are invited to communicate with M. P., 164a.

### Chronic Nephritis.

We do not undertake to give advice upon treatment in these columns. This is a matter upon which you must be guided by the medical practitioner dealing with the case. There is no reason, however, why you should not seek the opinion of a consultant. Discuss the matter with your doctor, and if you desire a second opinion tell him so frankly. We strongly urge you not to adopt the course you suggest.—"Father."

### *INSTITUTIONAL FACTS AND FIGURES.*

### Out-Patient Records.

An out-patient clerk sends us the following observations upon filing out-patient records :—

The Numerical System.

Possibly the method most generally in use for filing out-patient prescription forms is to separate them into bundles under the names of the medical officers attending the cases, and to place them in numerical order. As each new form is issued the patient is given a card, bearing the number of his or her prescription, for presentation on each subsequent visit. The system answers well enough so long as the patients remember to bring their cards, but, unfortunately, they cannot always be relied upon to do so, and consequently much time is lost in searching for papers the numbers of which are not known, an exceptionally difficult matter when the patient's last attendance is not of recent date. Having in view this defect, it is difficult to understand why the system is so popular.

The Up-to-date System.

An alphabetical system carried out on the lines of the American card-index system of vertical filing, with certain modifications, is in every way preferable, and obviates the disadvantage referred to. A drawer or box, preferably the former, should be allocated for the reception of the prescriptions of each member of the visiting staff, each containing a set of index cards which may be amplified by the introduction of cards bearing the second letter of the names of most frequent incidence, as well as the initial letters, in accordance with requirements. Between these cards the prescriptions can be very rapidly filed. The forms should be of convenient size—say large 8vo—preferably single sheets of stiff manilla, which is almost untearable. Generally speaking, the forms in use are much larger than they need be, a circumstance which adds to the difficulty of filing and preserving them in good condition. As the drawers become full the whole contents, including indices, should be transferred to inexpensive cardboard boxes, labelled with the medical officers' names and the commencing and closing dates. The forms which are from time to time abstracted from these boxes should be filed in the drawer in current use; the date of the patient's last attendance will then indicate the box or drawer in which the search for the prescription should be made, and the patient usually remembers this. The usual card may be issued to the patient and the date of each attendance stamped on the back, but it is of little consequence if this be lost.

### Supply of Drugs.

We do not advise you to contract for the supply of drugs and antiseptics in accordance with a schedule, for in our experience many items which are scheduled in drug contracts are never made use of; and, on the other hand, others are sometimes overlooked for which payment must necessarily be made outside the contract terms, if ordered. Certain drugs which are not subject to frequent market fluctuations can, of course, be advantageously contracted for if the quantity consumed is considerable. At an institution such as yours, however, we would suggest that you purchase in accordance with the manufacturers' monthly list, and arrange for a trade discount. Write to the various manufacturing chemists for their lists and ascertain what discounts they will be prepared to allow; by comparing the lists you should have no difficulty in making a selection. Be careful to purchase all proprietary articles direct from the manufacturers or their agents; you can effect a considerable saving in this way, for special hospital terms are often allowed. Whilst it is, of course, more economical to purchase drugs in the market at favourable opportunities, you would not find this course practicable, nor do your requirements warrant it.—" Hospital Secretary."

### Fire Appliances.

The fire appliances which comprise the equipment of most hospitals and public buildings are hydrants, hose, fire buckets, and chemical extinguishers. Yet whilst considerable expenditure may be incurred in installing and maintaining these, they may be rendered quite ineffective if some simple, but essential, article necessary for their application is not to hand. We have a striking illustration of this in connection with the fire which occurred at the Toronto Free Hospital. What was most urgently needed at this institution was an axe and a saw, but unfortunately owing to lack of foresight, these were stored in the basement, and were consequently inaccessible. Articles such as these might with advantage be located in various positions in the building and labelled "For fire only." Amongst other useful articles are ropes, ladders, stretchers, and blankets. Another suggestion which should prove extremely useful is the provision at nightfall of a woollen blanket and a bath-tub filled with water.

### *THE INSTITUTIONAL ARTIFICER.*

**QUESTIONS FOR JUNE.**

**1. Describe and, if possible, give a sketch or photograph of any home-made piece of hospital furniture or apparatus you have designed, or which you have found to serve a useful purpose at your institution.**

**2. Describe any defects you have found in your heating and hot-water system, and the steps you have taken to remedy them.**

*NOTE.—A minimum payment of FIVE SHILLINGS will be made for each descriptive note based upon the foregoing questions which is accepted for publication.*

RULES.

The following rules must be observed:—

1. Contributions must be written on one side of the paper. They must bear the name and address of the sender and be accompanied by coupon to be cut from the back cover (inside page) of the current issue of The Hospital. A pseudonym must be chosen if the name is not to be published.

2. They must be addressed to the Editor of The Hospital, 28 & 29 Southampton Street, Strand, London, W.C., so as to reach him before the end of the month, and must be marked in the left-hand corner "Institutional Artificer."

### Flushing Cistern.

The cistern to which you refer is probably Emanuel's A B C type. It consists of one large porcelain-enamelled casting containing two distinct flushing cisterns; it is practically noiseless in action and thoroughly reliable. The manufacturers are Messrs. A. Emanuel and Son, Ltd., George Street, Manchester Square, London.—" Sanitary."

### *THE INSTITUTIONAL HOUSEKEEPER.*

### Food Vessels.

You cannot satisfactorily serve the patients' dinners from the main kitchen in separate plates, even in a small hospital such as yours; for, however expeditiously these are distributed to the wards, many of them will be no more than lukewarm by the time the patients receive them. It is so important that food should reach the patients in warm and good condition that it appears to us to be false economy to consider the expense of proper food vessels. In most well-appointed institutions the food is distributed to the ward kitchens in metal containers holding trays for the separation of the different foods, and the water-jacketted type are obviously the best.—" Housekeeper."

### *EMPLOYMENT AND TRAINING.*

### Lady Sanitary Inspector.

A coupon should be enclosed with each inquiry; we cannot give postal replies. If you will refer to our last week's issue, you will find under the heading "Employment and Training" information respecting the appointment of lady sanitary inspectors. The necessary qualification is the certificate of the Sanitary Inspectors' Examination Board, particulars of which may be obtained upon application to the Secretary, 1 Adelaide Buildings, London Bridge, S.E.—" H. B. D."

### *EDITOR'S LETTER-BOX.*

The Editor is indebted for communications received from Miss L. Starey and Miss M. E. Garrard, which he has duly forwarded.

"M. P."—We thank you for your letter, and we have accordingly announced your need in these columns. We should be interested to hear the result of your application to the Maghull Home, which, as we indicated last week, receives epileptic women for 10s. 6d. a week.

The Editor will be glad to receive correspondence and to consider contributions upon all subjects relating to institutional work, and affecting the welfare of institutional officers.

### Editor's Notices.

**Contributions:**

Contributions should be written, or preferably typed, on one side of the paper only, and all articles sent in are accepted upon the distinct understanding that they are forwarded to The Hospital only.

The Editor cannot undertake to return MSS. not used, but when a stamped directed envelope is enclosed the MSS. may be returned if a special request is made.

Accepted articles and paragraphs of news will be paid for after publication at the scale rate.

**Address:**

To prevent delay all contributions and letters on editorial business must be addressed exclusively to the Editor, "The Hospital," 29 Southampton Street, Strand, London, W.C. It is important that this regulation shall be strictly observed.

**Correspondence:**

Correspondence on all subjects is invited. The name and address of correspondents must be given as a guarantee of good faith, but not necessarily for publication.

**Special Articles:**

Special articles are invited, and questions, inquiries, and paragraphs upon all matters relating to the work, administration, and management of general, special, mental, fever, cottage and convalescent hospitals, sanatoria, homes, institutions, societies and organisations for the treatment or care of the sick, injured and dependants of all classes. Every matter affecting the interests and welfare of the staff of all grades working in these institutions will receive special consideration.

**Administrative Medicine, Research and Items of News:**

Special terms will be paid for approved articles on subjects in this wide field by experts who have made some section of it their special study and interest. We wish to give prominence to every movement tending to advance accuracy of diagnosis, efficiency in treatment, and the development of modern methods to eradicate disease and promote the welfare of the suffering.

**A Bureau of Information:**

We invite inquiries and applications for information and help in providing for that numerous class of sufferers who require special care or house-room which they cannot obtain in their own homes or secure for themselves. **We cannot, however, prescribe, or recommend practitioners.**

**Books for Review:**

Publishers are particularly requested to send advance proofs of any new books of importance whenever possible, as the Editor has made arrangements to publish immediate reviews on a new plan.

**Photographs, Plans, Blocks, and Illustrations:**

It is requested that wherever possible MSS. may be accompanied by illustrations in any of the above forms. The name of the sender and of the article to which it belongs should be written on the back of each photograph, drawing, or block for purposes of identification.

**Local Papers:**

Newspapers containing reports or news paragraphs **should be marked** and addressed to the Sub-Editor.

**The Coupon System:**

A coupon will be found at the bottom of the third inside page of the cover each week. This coupon must be attached to every question or inquiry to which an answer is desired in The Hospital.

### Interesting Facts.

**For the Institutional Worker**

This Special Supplement affords exceptional facilities to those seeking appointments in any grade or department of the Institutional, Health, and Sanitary Services. In this section a large number of vacancies in Hospitals, Infirmaries, and Institutions of every description throughout the United Kingdom are announced every week. Should you not find a post suited to your needs we would remind you that a column is reserved to those seeking re-engagement, and an announcement of twenty-one words, costing 1s., in this portion of the Journal will enable you to bring your requirements prominently before the Executive Officials of every class of Institution, as well as those responsible for Poor-Law and Municipal administration.

**For Institution Authorities:**

The officials controlling institutions will also find The Hospital of the greatest practical utility when a vacancy occurs upon their staffs, as an announcement in its columns secures the widest publicity among those trained to Institutional Work, and through its agency it is possible to obtain the most efficient officers in every section of Institutional Life. In these circumstances particular attention is drawn to the fact that Special Rates are allowed to those Institutions that agree to send all their public notices for insertion in this section of The Hospital.

### Manager's Notices.

**Letters relating to the publication, sale, and advertising departments of "The Hospital" must be addressed to The Manager, "The Hospital," The Hospital Building, 28 and 29 Southampton Street, London, W.C.**

**Subscriptions** may begin at any time, and are payable in advance. Cheques and Post-Office Orders should be crossed London County and Westminster Bank, Covent Garden Branch, and made payable to the Manager of The Hospital.

Rates of Subscription (payable in advance).

UNITED KINGDOM.

| | | |
|---|---|---|
| Three Months (including Postage) | ... ... ... ... | 2s. 0d. |
| Six Months | do. ... ... ... ... | 4s. 0d. |
| Twelve Months | do. ... ... ... ... | 6s. 6d. |

FOREIGN AND COLONIAL.

| | | |
|---|---|---|
| Three Months (including Postage) | ... ... ... ... | 2s. 6d. |
| Six Months | do. ... ... ... ... | 5s. 0d. |
| Twelve Months | do. ... ... ... ... | 8s. 8d. |

**Advertisements:**

To ensure insertion, all advertisements for The Hospital must reach the Manager not later than Wednesday morning in each week. For scale of charges see pages v. and 4.

**Remittances:**

**It is especially requested that remittances be made by Cheque or Postal Order, and in halfpenny stamps only when the amount is under sixpence.**

